# Time dependent effect of cold ischemia on the phosphoproteome and protein kinase activity in fresh-frozen colorectal cancer tissue obtained from patients

**DOI:** 10.1186/s12014-020-09306-6

**Published:** 2021-02-18

**Authors:** Tineke E. Buffart, Rosanne A. H. M. van den Oord, Adriënne van den Berg, Riet Hilhorst, Niek Bastiaensen, Hans F. M. Pruijt, Adriaan van den Brule, Peet Nooijen, Mariette Labots, Richard R. de Goeij-de Haas, Henk Dekker, Sander R. Piersma, Thang V. Pham, Theo van der Leij, Rik de Wijn, Rob Ruijtenbeek, Connie R. Jiménez, Henk M. W. Verheul

**Affiliations:** 1 Department of Medical Oncology, Amsterdam UMC, VU University Medical Center, Amsterdam, The Netherlands; 2 Department of Gastrointestinal Oncology, Netherlands Cancer Institute, Amsterdam, The Netherlands; 3 Department of Medical Oncology, Jeroen Bosch Hospital, ‘S-Hertogenbosch, The Netherlands; 4 PamGene International BV, ‘S-Hertogenbosch, The Netherlands; 5 Laboratory of Molecular Diagnostics, Jeroen Bosch Hospital, ‘S-Hertogenbosch, The Netherlands; 6 Department of Pathology, Jeroen Bosch Hospital, ‘S-Hertogenbosch, The Netherlands; 7 GenMab BV, Utrecht, The Netherlands; 8 Department of Medical Oncology, Radboud University Medical Center, Geert Grooteplein 10, 6525GA Nijmegen, The Netherlands

**Keywords:** Protein kinase, Mass spectrometry, Phosphoproteomics, Peptide microarray, Cold ischemia, Cancer

## Abstract

**Background:**

Based on their potential to analyze aberrant cellular signaling in relation to biological function, kinase activity profiling in tumor biopsies by peptide microarrays and mass spectrometry-based phosphoproteomics may guide selection of protein kinase inhibitors in patients with cancer. Variable tissue handling procedures in clinical practice may influence protein phosphorylation status and kinase activity and therewith may hamper biomarker discovery. Here, the effect of cold ischemia time (CIT) on the stability of kinase activity and protein phosphorylation status in fresh-frozen clinical tissue samples was studied using peptide microarrays and mass spectrometry-based phosphoproteomics.

**Methods:**

Biopsies of colorectal cancer resection specimens from five patients were collected and snap frozen immediately after surgery and at 6 additional time points between 0 and 180 min of CIT. Kinase activity profiling was performed for all samples using a peptide microarray. MS-based global phosphoproteomics was performed in tumors from 3 patients at 4 time points. Statistical and cluster analyses were performed to analyze changes in kinase activity and phosphoproteome resulting from CIT.

**Results:**

Unsupervised cluster analysis of kinase activity and phosphoproteome data revealed that samples from the same patients cluster together. Continuous ANOVA analysis of all 7 time points for 5 patient samples resulted in 4 peptides out of 210 (2%) with significantly (p < 0.01 and fold change > 2) altered signal intensity in time. In 4 out of 5 patients tumor kinase activity was stable with CIT. MS-based phosphoproteomics resulted in the detection of 10,488 different phosphopeptides with on average 6044 phosphopeptides per tumor sample. 2715 phosphopeptides were detected in all samples at time point 0, of which 90 (3.3%) phosphopeptides showed significant changes in intensity with CIT (p < 0.01). Only two phosphopeptides were significantly changed in all time points, including one peptide (PKP3) with a fold change > 2.

**Conclusions:**

The vast majority of the phosphoproteome as well as the activity of protein kinases in colorectal cancer resection tissue is stable up to 180 min of CIT and reflects tumor characteristics. However, specific changes in kinase activity with increasing CIT were observed. Therefore, stringent tissue collection procedures are advised to minimize changes in kinase activity during CIT.

## Background

Protein kinases are key regulators of cellular processes such as cell migration, proliferation, differentiation and survival [1]. Alterations in protein kinase activity can disrupt normal cellular signaling resulting in malignant transformation of cells [2]. Inhibiting the function of deregulated kinases can inhibit proliferation of cancer cells. In the past decades an increasing number of protein kinase inhibitors (PKIs) have become clinically available for treatment of various cancer types. When considering molecular profiling strategies to predict efficacy of targeted therapies in individual patients, ideally techniques considering the methods of action and level of activity should be used. Essentially, all available targeted therapies are directed against signaling molecules rather than genes. Gene expression studies and protein abundance may provide useful information, but a better-alternative strategy would be to analyze networks that underlie cancer development, progression and therapeutic resistance at both a personal and systems-wide level. Such techniques include peptide microarray-based kinase activity profiling and phosphoproteomics, adding unique information on the complex signaling pathways altered in cancer cells. Additionally, they may contribute to the discovery of potential new biomarkers and novel treatment targets [3–6].

Still, one of the challenges for the application of such technologies is the requirement of fresh frozen tumor tissue samples since formalin-fixed paraffin-embedded (FFPE) material is not suitable due to the fixation procedure affecting kinase activity and the protein phosphostatus [7]. In clinical sample handling and tissue processing, the freezing method and storage of tumor tissue may affect cellular signaling and protein phosphorylation levels. Especially the time from surgical tumor resection to freezing, referred to as cold ischemia time (CIT), can affect kinase activity and protein phosphostatus. CIT could have implications for discovery of biomarkers using fresh frozen material [8].

Previous mass spectrometry (MS)-based studies reported that the effect of CIT on protein phosphorylation varies from stable to unpredictable changes in phosphosites [9–12]. The effect of CIT on kinase activity profiles using peptide microarrays has not been investigated before. Here we investigate the influence of cold ischemia on cellular signaling activities using colorectal cancer (CRC) tissue samples obtained in a clinical setting.

## Materials and methods

### Colorectal cancer tissue collection

Colorectal cancer (CRC) tissue samples were collected from five patients (Patient 1–5) who underwent primary surgery at the Jeroen Bosch Hospital (‘s-Hertogenbosch, The Netherlands) without any prior treatment for CRC. All tumors were evaluated for histopathological tumor type and percentage of tumor cells by a pathologist (PN). The first biopsy (t = 0) was obtained immediately after surgical resection and snap-frozen in the operating room. The resection specimen was kept at room temperature and subsequent biopsies were taken and snap-frozen after 30, 60, 90, 120, 150 and 180 min of CIT. All patients provided informed consent after approval for the study was obtained from the institutional review board of the Jeroen Bosch Hospital.

### Lysis of sample, peptide microarray (PamChip®) assay and data analysis

From each biopsy one cryosection of 5 µm was made and stained with hematoxylin and eosin (H&E) for evaluation of tumor content, followed by three cryosections of 60 µm which were lysed separately (sample preparation replicates) in M-PER lysis buffer (1section/100 µl, Mammalian Protein Extraction Reagent, Thermo Fischer Scientific, MA, USA) containing Protease Inhibitor Cocktail (Thermo Fischer Scientific, MA, USA) and Phosphatase Inhibitor Cocktail (Thermo Fischer Scientific, MA, USA). After centrifugation (10 min, 4 °C, 10,000× *g*) the supernatant was collected, aliquoted and frozen at −80 °C. The protein concentration was determined using the Bradford Lowry Assay (Thermo Fischer Scientific, MA, USA) according to the manufacturer’s instructions.

Protein tyrosine (PTK) and serine/threonine (STK) kinase activity of the lysates of the biopsies was analyzed using tyrosine or serine/threonine peptide microarrays (PamChip®) measured in a PamStation 96 according to the instructions of the manufacturer (PamGene International BV, ‘s-Hertogenbosch, The Netherlands). Each array contains 142 peptides derived from known tyrosine or serine/threonine phosphorylation sites in human proteins and 2 control peptides.

For every time point, two technical and three sample preparation replicates were assayed. For the sample preparation replicates, consecutive sections of the same biopsy were lysed separately.

Tyrosine kinase activity was determined in the lysates using 5 μg of total protein of each sample in a final volume of 40 μl of protein kinase buffer (10 mM MgCl_2_, 1 mM EDTA, 2 mM DTT, and 0.01% Brij 35 in 50 mM Tris/HCl (pH 7.5)), supplemented with 0.01% BSA, 10 mM DTT and PTK additive (PamGene International BV), 12.5 μg/ml fluorescein-labeled anti-phosphotyrosine antibody PY20 (Exalpha, Maynard, MA, USA) and 400 μM ATP (Sigma-Aldrich, Zwijndrecht, The Netherlands). Incubations without ATP were performed as a control for non-specific binding to the peptides.

Prior to application of the sample, the peptide microarrays were blocked with 2% bovine serum albumin (BSA) (Fraction V, Calbiochem) and washed three times with protein kinase buffer. During a 60-min incubation at 30 °C, the sample mix was pumped up and down (once per minute) through the porous array surface. Real time images of the arrays, showing the binding of the fluorescein-labeled PY20 antibody to peptides that are phosphorylated by kinases present in the CRC sample, were taken automatically every 5 min and after 60 min of incubation after washing the arrays. Serine/threonine kinase activity was determined in the lysate by incubating 1 µg of protein/array for 60 min in protein kinase buffer, supplemented with 0.01% BSA, 400 µM ATP and anti-phospho serine/threonine antibodies (PamGene International BV, ‘s-Hertogenbosch, the Netherlands). After washing a secondary antibody was applied and images were taken at 5-min intervals for 30 min, and at multiple exposure times after washing the arrays. Signal intensity on each spot at each time point was quantified with BioNavigator software 6.2 (PamGene International BV), as described previously [13].

Arrays that showed defects upon visual inspection were excluded. For each peptide spot the local background around that spot was subtracted from the signal intensity in the spot. Peptides that showed an increase in signal in time, as measure for *bona fide* ATP-dependent phosphorylation, in at least 20% of the arrays were included in the PTK and STK analysis.

Visualization and statistical analysis were performed using BioNavigator software 6.2. An analysis of variance (ANOVA) test with time till CIT as continuous factor was performed to test which array peptide phosphorylation levels were changing significantly in time. The analysis for the combined data was performed with a mixed-effects model including the factor “time” as fixed and the factor “patient” as random. Peptides were considered significantly differential when the p-value was < 0.01.

### Protein isolation, nano liquid chromatography-tandem mass spectrometry (nanoLC-MS/MS) and data analysis

For MS-based phosphoproteomics biopsies were collected from three of the five patients at four time points (t = 0, 60, 120 and 180 min of CIT) similar to the peptide array experiment. Cryosections of 5 µm were stained with H&E for evaluation, followed by sixty 10 µm sections for protein isolation. These 10 µm sections were lysed in 20 mM HEPES (pH 8.0) buffer containing 9 M urea, 1 mM Na_3_VO_4_ (orthovanadate), 2.5 mM Na_4_P_2_O_7_ (pyrophosphate), and 1 mM Na_2_C_3_H_7_PO_6_ (ß-glycerophosphate). After lysis, the protein concentration was determined using the bicinchoninic acid (BCA) method according to the manufacturer’s instructions (Thermo Fischer Scientific, Rockford, IL). Digestion of the lysates and global phosphopeptide enrichment using Titanium dioxide (TiO_2_) beads were performed as previously described [14]. Briefly, tissue lysates were reduced in 4.5 mM DTT for 30 min at 55 °C, cooled to room temperature and alkylated in 11 mM iodoacetamide for 15 min in the dark. Subsequently, tissue lysates were diluted in 20 mM HEPES pH8.0 to reduce the urea concentration to 2 M and digested overnight with trypsin (Promega, Madison, WI, US) with an enzyme protein ratio of 1:50 (w/w).

### Phosphopeptide enrichment

For each CRC tissue sample, 500 µg of tryptic lysate digests were acidified by adding TFA to a final concentration of 1%, and incubated on ice for 15 min. Samples were desalted with 30 mg OASIS HLB cartridges (Waters Corporation, Milford, MA, US), previously activated in 100% ACN and equilibrated in 0.1% TFA. Briefly, peptides were loaded in the cartridge, washed in 0.1% TFA, and eluted in 0.1% TFA/80% ACN solution. Subsequently, desalted peptides were diluted 1:1 with lactic acid solution (0.3 g/ml lactic acid, 0.07% TFA, 53% ACN). For phosphopeptide capture, 2.5 mg of TiO_2_ beads (GL sciences, 10 µm) were packed in a 200-µl pipette tip fitted with a 1 mm needle punch of C8 material at the narrow end. Tips containing the TiO_2_ bed were washed with 200 µl of 0.1% TFA/80% ACN and equilibrated with 200 µl of 300 mM acid lactic solution. Desalted peptides were loaded in the tips in 5 cycles of 200 µl of peptide mixture and centrifuged at 1500×*g* for 4 min. The TiO_2_ bed with bound phosphopeptides was then washed, firstly with 200 µl lactic acid solution, and secondly with 200 µl 0.1% TFA/80% ACN. All steps were performed by centrifugation at 1500×*g* for 4 min. Phosphopeptides were eluted in two steps with 50 µl 0.5% piperidine (Thermo Fisher Scientific) and 50 µl 5% piperidine, and subsequently quenched in 100 µl 20% H_3_PO_4_. Phosphopeptides were desalted using 200-µl pipette tips fitted with a 1 mm-needle punch of SDB-XC SPE material at the narrow end, which was previously washed with 20 µl 0.1% TFA/80% ACN and equilibrated with 20 µl 0.1% TFA. Phosphopeptides were loaded and centrifuged for 3 min at 1000×*g*. SDB-XC beds were then washed with 20 µl of 0.1% TFA, and desalted phosphopeptides were eluted with 20 µl of 0.1% TFA/80% ACN. Phosphopeptides were dried in a vacuum centrifuge and dissolved in 20 µl 0.5% TFA/4% ACN prior to injection.

### LC–MS/MS

Peptides were separated by an Ultimate 3000 nanoLC-MS/MS system (Dionex LC-Packings, Amsterdam, The Netherlands) equipped with a 40 cm × 75 μm ID fused silica column custom packed with 1.9 μm 120 Å ReproSil Pur C18 aqua (Dr Maisch GMBH, Ammerbuch-Entringen, Germany). After injection, peptides were trapped at 6 μl/min on a 10 mm × 100 μm ID trap column packed with 5 μm 120 Å ReproSil Pur C18 aqua at 2% buffer B (buffer A: 0.5% acetic acid (Fischer Scientific), buffer B: 80% ACN, 0.5% acetic acid) and separated at 300 nl/min in a 10–40% buffer B gradient in 90 min (120 min inject-to-inject). Eluting peptides were ionized at a potential of + 2 kVa into a Q Exactive mass spectrometer (Thermo Fisher, Bremen, Germany). Intact masses were measured at resolution 70.000 (at m/z 200) in the orbitrap using an AGC target value of 3E6 charges. The top 10 peptide signals (charge-states 2 + and higher) were submitted to MS/MS in the HCD (higher-energy collision) cell (1.6 amu isolation width, 25% normalized collision energy). MS/MS spectra were acquired at resolution 17.500 (at m/z 200) in the orbitrap using an AGC target value of 2E5 charges and an underfill ratio of 0.1%. Dynamic exclusion was applied with a repeat count of 1 and an exclusion time of 30 s.

MS/MS spectra were searched against the Uniprot human reference proteome FASTA file (release January 2014, 61,552 entries, NO fragments) using MaxQuant 1.4.1.2.[14]. The default MaxQuant settings for Orbitrap MS were used. The maximum allowed error in MaxQuant search was 4.5 ppm for MS1 and 20 ppm for MS2. The phosphosites were class I (localization probability > 0.75) and phosphosite scores were filtered to an FDR of 1% (default MaxQuant setting). Phosphopeptides were quantified by their extracted ion chromatograms (‘Intensity’ in MaxQuant). For each sample the phosphopeptide intensities were normalized on the median intensity of all identified phosphopeptides in the sample (‘Intensity’ from the MaxQuant evidence table). Normalized phosphopeptide intensities were used for calculations of fold changes of each peptide between each time point compared to time point t = 0. Fold changes of > 2 were considered biologically relevant. *P* values were calculated using a linear model test. Phosphopeptides were considered significantly different when the p-value was < 0.01 after correction for multiple testing using the Benjamini–Hochberg method. Peptides with missing values were excluded from subsequent statistical analysis. Unsupervised cluster analysis was performed using R version 3.4.2 (R Development Core Team).

## Results

### Sample characteristics

From the CRC tissue samples of the five patients included in this study, four were histologically classified as adenocarcinoma and one tumor had a mucinous type histology. The mean age of the patients was 62 (49–74) years and four of the five patients were male. The mean percentage of tumor cells was 43% (5–95%) in the samples included in the peptide array and 51% (25–70%) in the samples included in the MS-based phosphoproteomics. Patient and tumor characteristics, including tumor cell percentages, are shown in Table 1.

**Table 1:** Overview of patient and tumor characteristics

Patient	Time-points (min)	Gender	Age	Location	Tumor type	differentiation	Tumor % Peptide array	Tumor % LC–MS–MS
1	0	Male	62	Caecum	Adenocarcinoma	Good/intermediate	50%	50%
	30						80%	
	60						95%	70%
	90						95%	
	120						60%	70%
	150						80%	
	180						70%	70%
2	0	Male	60	Rectum	Mucinous		20%	
	30						7.5%	
	60						5%	
	90						10%	
	120						10%	
	150						20%	
	180						20%	
3	0	Male	74	Sigmoid	Adenocarcinoma	Good/intermediate	50%	
	30						25%	
	60						25%	
	90						25%	
	120						25%	
	150						25%	
	180						37.5%	
4	0	Female	49	Caecum	Adenocarcinoma	Good/intermediate	75%	70%
	30						50%	
	60						40%	55%
	90						30%	
	120						20%	25%
	150						70%	
	180						70%	35%
5	0	Male	64	Ascending colon	Adenocarcinoma	Good/intermediate	25%	30%
	30						50%	
	60						50%	60%
	90						50%	
	120						50%	40%
	150						50%	
	180						50%	40%

Patient (1–5) and tumor characteristics of timepoint 0 and 6 timepoints between 30–180 min of CIT are listed, including tumor percentage (%) of each of the analyzed samples for both the kinase peptide array and with nano liquid chromatography-tandem mass spectrometry-based phospoproteomics (LC–MS–MS)

### The effect of cold ischemia time on protein kinase activity

After selection of peptides showing ATP dependent signals in at least 20% of the arrays 93 out of 142 PTK peptides and 117 out of 142 STK peptides were retained for further analysis. To test the reproducibility of the workflow technical replicates and sample preparation replicates were analyzed for each time point. For technical replicates (n = 2) the coefficient of variation (CV) was 19% and 14% for the PTK and STK signals respectively. The CV’s of the sample preparation replicates (n = 3) were 19% (range 14–22%) for the PTK signals and 15% (range 10–22%) for the STK signals, indicating that the workflow is reproducible (Additional files 1, 2: Figure S1).

**Fig. 1:**
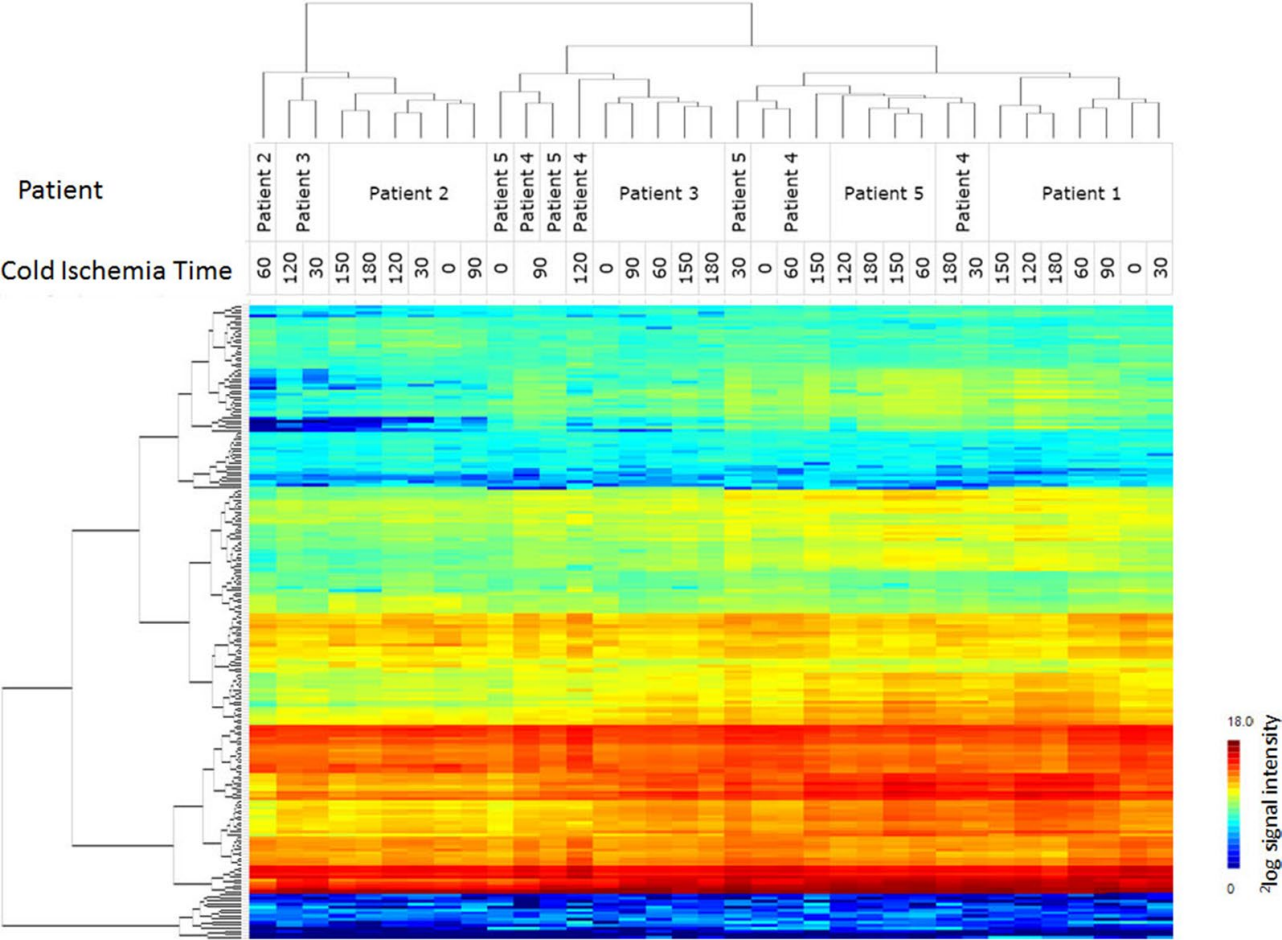
Unsupervised hierarchical cluster analysis of tyrosine and serine/threonine kinase activity profiles**.** Unsupervised cluster analyses of the phosphorylation profiles of the tumor samples from five patients on time points 0, 30, 60, 90, 120, 150 and 180 min of CIT. The different tumor samples included in the study are represented on the X-axis and ^2^log signal intensity of each kinase is represented on the Y-axis. Tumor samples from the same patient largely cluster together indicating that changes in kinase activity are more pronounced between tumors of different patients rather than based on differences in CIT

Unsupervised cluster analysis of the signal intensities was performed with the PTK and STK peptide microarray data combined to visualize differences between the tumors from the five patients at different time points (Fig. 1, for details see Additional file 3: Figure S2). Tumor samples from the same patient largely cluster together indicating that interpatient variability in kinase activity is larger than the variation between samples during CIT.

**Fig. 2:**
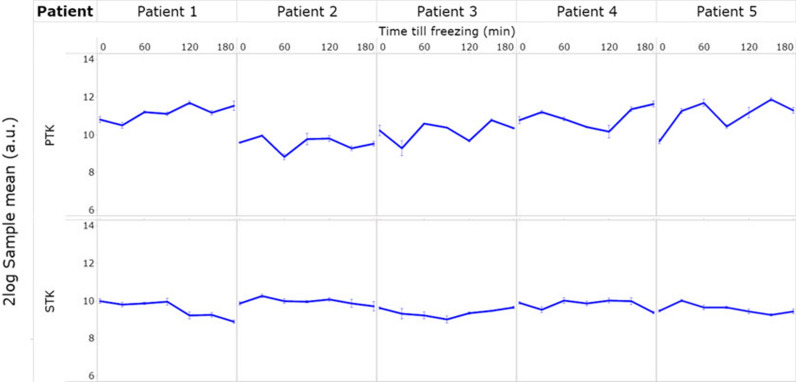
Mean protein tyrosine kinase (PTK) and serine/threonine kinase (STK) activity with CIT (peptide microarray). The mean signal intensity of all peptides at seven time points (T = 0, 30, 60, 90, 120, 150 and 180 min of CIT) for the tumors of all five patients (patient 1–5) for PTK and STK are represented (X-axis). The mean value for all samples is represented in a ^2^log scale (Y-axis). For each peptide values were the average of six datapoints. First, the median signal minus background values per array for all the technical replicates were calculated and then the average for the sample preparation replicates was calculated. Subsequently the mean of all peptides was calculated

**Fig. 3:**
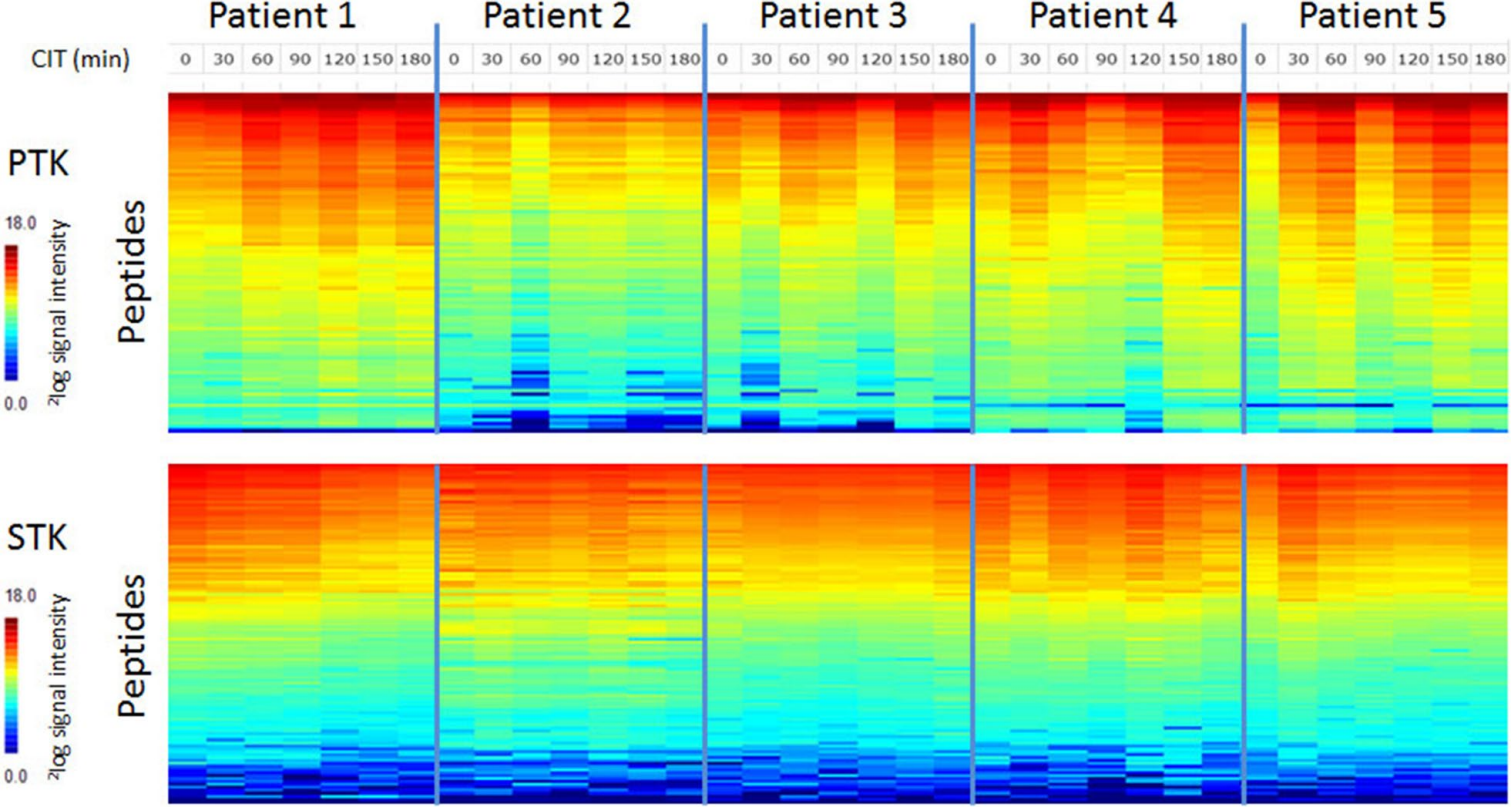
Heatmap of protein tyrosine kinase (PTK) and serine/threonine kinase (STK) phosphorylation. Heatmap of ^2^log transformed mean signal intensities for PTK and STK activity (X-axis) profiles of the tumors of patient 1–5 as function of CIT for the time points 0, 30, 60, 90, 120, 150 and 180 min (Y-axis). Only peptides that passed quality control were included. Peptides are ordered by signal intensity

To analyze whether kinase activity changes with CIT, for each tumor the mean kinase activity was calculated for each time point. Although fluctuation was observed in the mean kinase activity between the different time points, no obvious structural increase or decrease in kinase activity was detected, except for patient 1 where signal intensity increased with CIT for PTK, but decreased for STK (Fig. 2, Additional file 3: Table S1). Overall fluctuations were less in STK activity than in PTK activity. A heatmap with signal intensities represented as the mean signal for each sample per tumor is shown in Fig. 3.

**Fig. 4:**
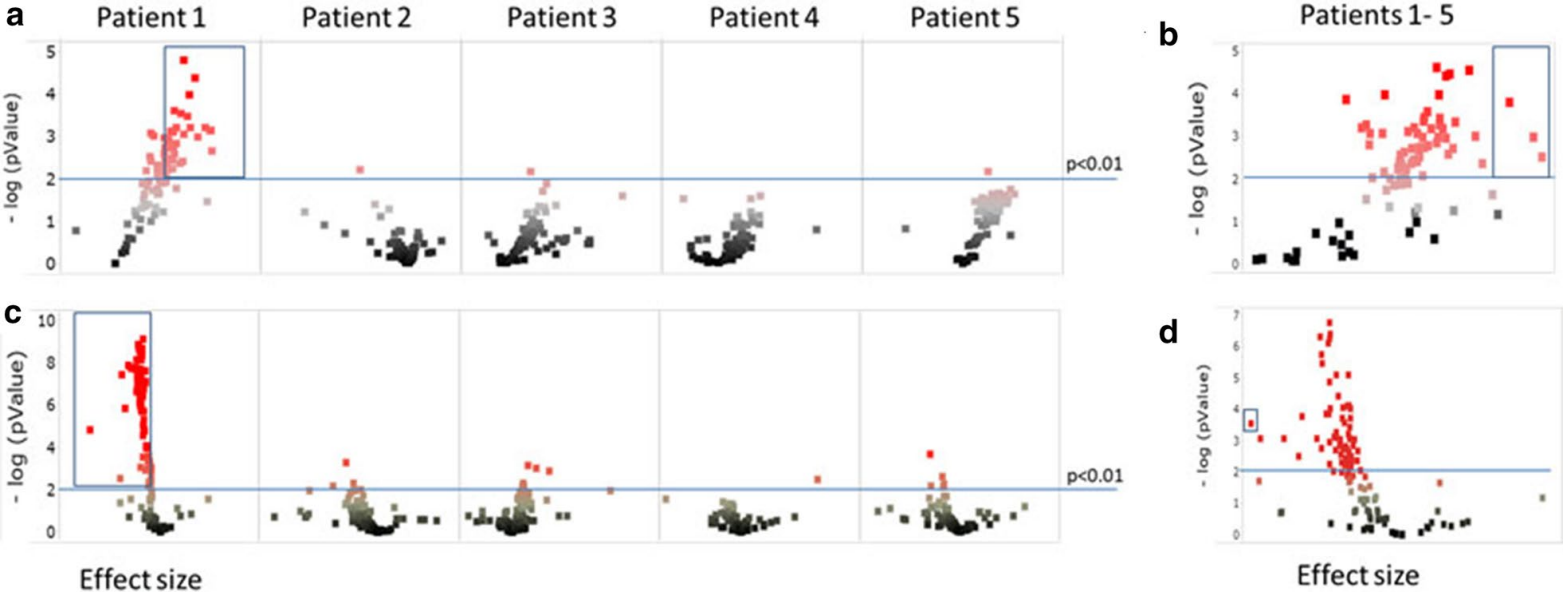
Correlation of signal intensity with CIT. Statistical analysis of the correlation of signal intensity with time till freezing analyzed per tumor (**a** PTK and **c** STK) and for all tumors combined (**b** PTK and **d** STK) represented in Volcano plots. The -log p-values (Y-axis) are plotted as function of effect size (X-axis). Peptides are colored according to p-values. Peptides with a p-value < 0.01 and effect size > 2 are boxed. In total 33 (35.5%) peptides (box in **a**, patient 1) and 3 (3%) peptides (box in **b**) of the peptides were phosphorylated with a significant (p < 0.01) and ≥ twofold increased signal after 180 min of CIT. For STK 62 (53%, box in **c**, patient 1) and 1 (0.9%, box in **d**) peptides showed a significant (p < 0.01) and ≥ twofold decreased signal intensities with 180 min of CIT

For the tumor tissues of the patients 2–5 none, one or two peptides were significantly (p < 0.01) changed with a fold change > 2 over 180 min of CIT (Fig. 4a and c, Additional file 3: Table S1). No overlap was found between the sets of significantly changing peptides for these four different tumors. However, in patient 1, 56 (60.2%) PTK and 79 (67.5%) STK peptides respectively showed a significantly increased and decreased phosphorylation signal with CIT, including 33 (35.5%) PTK and 62 (53.0%) STK peptides with a fold change > 2 after 180 min of CIT (Additional file 3: Table S1). To investigate changes common to all tumor samples, data was combined in a mixed model analysis resulting in 60 (65.2%) and 62 (53.0%) peptides for the PTK and STK data sets, respectively, which were phosphorylated with a significantly increased (PTK) or decreased (STK) signal in time (p < 0.01) (Fig. 4b and d). Of these, three PTK (Annexin A2 (UniProt accession P07355; sequence: HSTPPSAYGSVKA), PDGFRB (UniProt accession P09619; sequence VSSDGHEYIYVDP) and STAT4 (UniProt accession Q14765l; sequence PSDLLPMSPSVYA)) and one STK (Phospholemman precursor (UniProt accession O00168; sequence EEGTFRSSIRRLS)) peptide showed a fold change > 2 after 180 min of CIT (Additional file 3: Table S1).

### The effect of cold ischemia time on the phosphoproteome

**Fig. 5:**
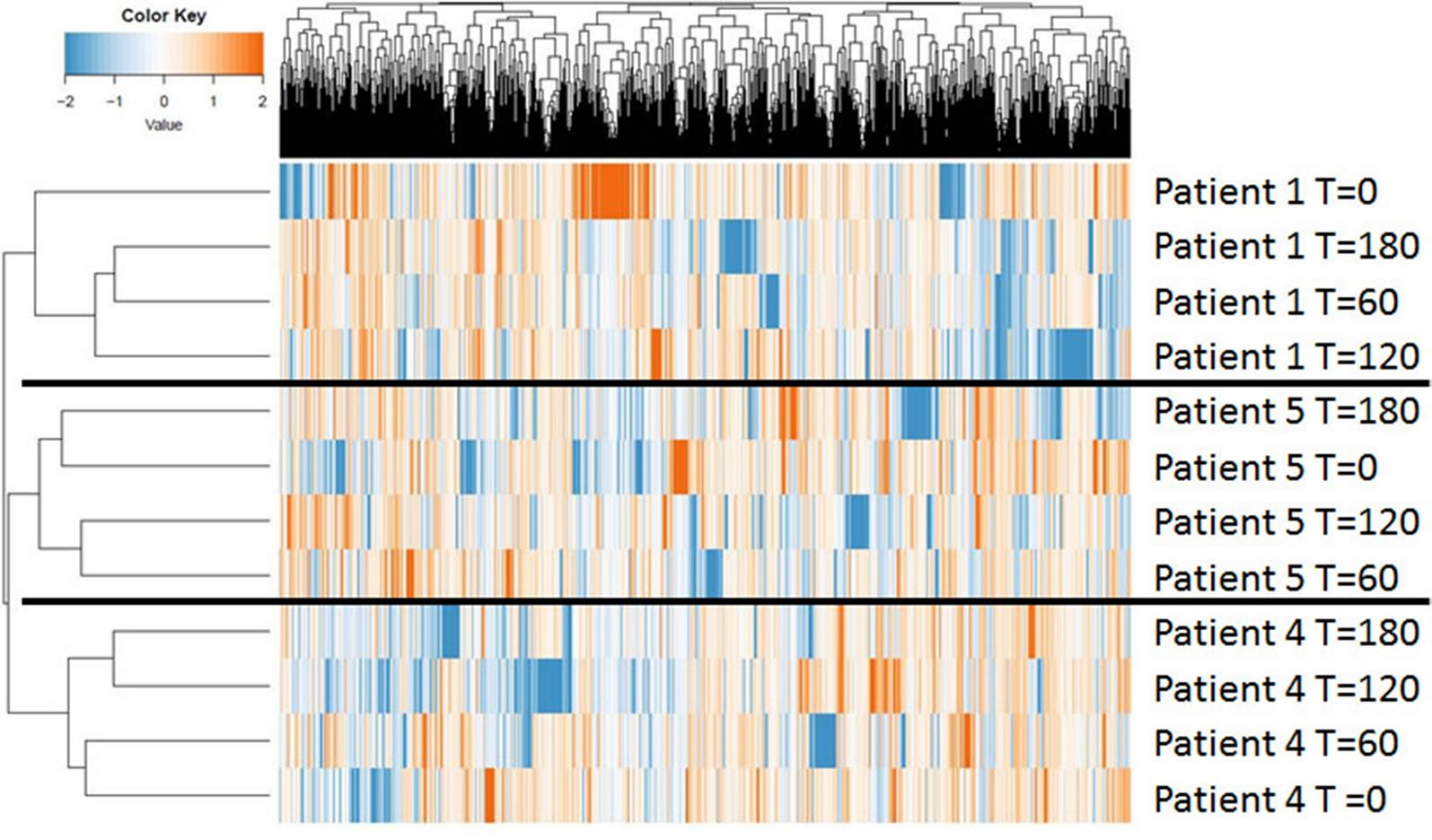
Unsupervised hierarchical clustering of phosphopeptides. Unsupervised cluster analysis of the 2715 phosphopeptides detected in all t = 0 samples. Tumor samples are represented on the X-axis and the signal intensities of the phosphopeptides are represented on the Y-axis. Unsupervised clustering of three different tumors at four different time points shows a clear clustering of the samples per tumor indicating that cold ischemia does not obscure interpatient differences in the phosphoproteome

**Fig. 6:**
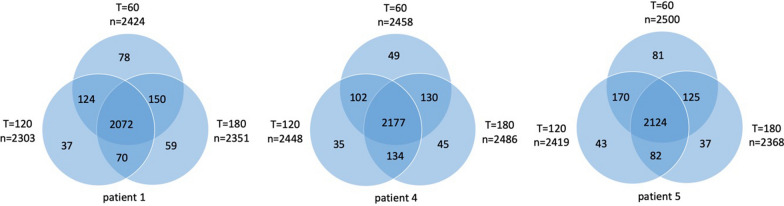
The number of phosphopeptides during CIT. For each patient (patient 1, 4 and 5) the number of phosphopeptides detected at each timepoint (t = 60, t = 120 and t = 180 min of CIT) compared to the 2715 phospopeptides detected at all three T = 0 timepoints. Numbers are represented in a venndiagram of each patient

MS-based global phosphoproteomics revealed 10,488 different phosphopeptides with on average 6044 phosphopeptides per tumor sample. In total, 1639 peptides were detected in all samples and 2715 phosphopeptides were detected in all three samples at t = 0. Unsupervised cluster analysis of intensities of the phosphopeptides present at t = 0, across all time points, showed a clear clustering of the samples per patient implying that the differences between the time points are smaller than the differences between the patients and supports that CIT does not obscure interpatient differences in phosphoproteomic profiles (Fig. 5). An overview of the number of overlapping and different phosphopeptides with the various time points during CIT of each patient separately is represented in Fig. 6.

**Fig. 7:**
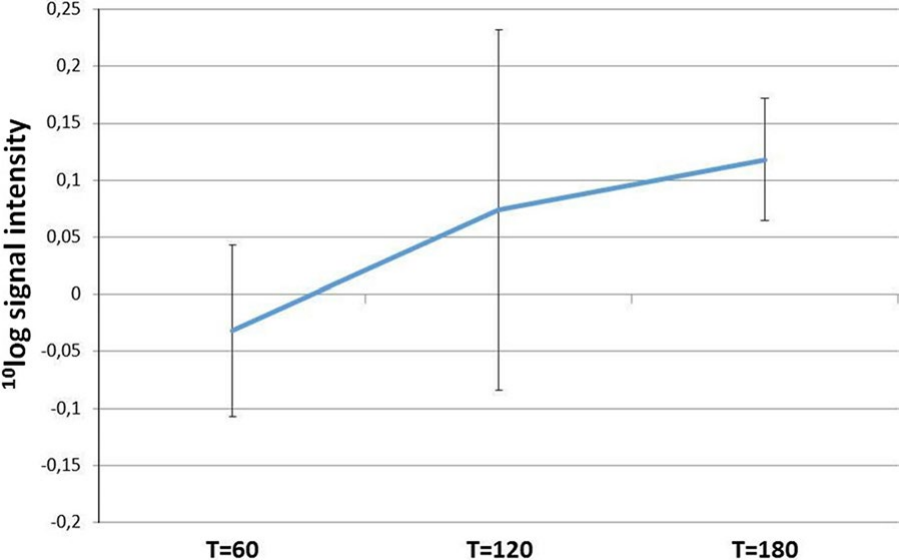
Signal intensities of peptides changing with CIT. Overview of mean (line) and standard deviation (error bars) of the signal intensities of the 90 peptides significantly changing with CIT compared to T = 0 (Y-axis). The different times of CIT compared to t = 0 (t = 60, 120 and 180 min thereafter) are represented on the X-axis

Statistical analysis (one-way ANOVA) resulted in 90 peptides (3.3%), including MAPK1 (p-peptide sequence: VADPDHDHTGFLTEYVATR) belonging to the stress-activated MAP kinase (MAPK) pathway, which were significantly changing with CIT compared to t = 0 (p < 0.01). No other functional proteins were directly involved in stress response. An overview of the mean signal intensities of these significant peptides of each time point including the standard deviation is represented in Fig. 7.

In addition to comparing changes in phosphopeptide intensities with CIT compared to t = 0, the effect of CIT in the phosphoproteome was analyzed per time point combining data for all three patients and all four time points. Between time point 0 and 60 min of cold ischemia a total of 28 (0.46%) phosphopeptides were significantly different (p < 0.01) with 18 upregulated peptides and 10 downregulated peptides. Of these, 15 and 4 peptides showed a fold change > 2 and > 3 respectively (Table 2; Additional file 3: Table S2). Between time point 0 and 120 min a total of 48 (0.79%) phosphopeptides were significantly different with 23 upregulated and 25 downregulated peptides. Of these 31 and 16 peptides were significantly different with a fold change of > 2 and > 3 respectively (Table 2; Additional file 3: Table S3). Between time point 0 and 180 min 52 (0.86%) phosphopeptides, with 31 upregulated and 21 downregulated peptides, were significantly different, including 23 peptides with a fold change > 2 and 13 peptides with a fold change > 3 (Table 2; Additional file 3: Table S4). Of all significant peptides 2 peptides, including 1 upregulated (mapped at protein SRRM2; p-peptide sequence TPAAAAAMNLASPR (T0-T60), MGQAPSQSLLPPAQDQPRSPVPSAFSDQSR (T0-T120), SRTPPSAPSQSR and SSRSSPELTR (T0-T180)) and 1 downregulated peptide (mapped at protein PKP3; p-peptide sequence ADYDTLSLR (T0-T60), ADYDTLSLRSLR (T0-T120), SAVDLSCSR (T0-T180)), were overlapping between the different time points (Additional file 3: Tables S2–S4).

**Table 2:** Overview of the number of detected phosphopeptides by MS-based phosphoproteomics in colorectal cancer tissue samples, including the number of phosphopeptides that significantly change with CIT (p < 0.01)

Number of patients	3								
Number of samples	12								
Total number of peptides	10,488								
Average number of peptides per sample	6,044			FC > 2			FC > 3		
T0 versus T60	28 (0.46%)	Up	18	15 (0.25%)	Up	10	4 (0.07%)	Up	3
		Down	10		Down	5		Down	1
T0 versus T120	48 (0.79%)	Up	23	31 (0.51%)	Up	15	16 (0.26%)	Up	7
		Down	25		Down	16		Down	9
T0 *versus* T180	52 (0.86%)	Up	31	23 (0.38%)	Up	15	13 (0.22%)	Up	7
		Down	21		Down	8		Down	6
Overlap between time points	2 (0.03%)	Up	1	1 (0.02%)	Up	0	1 (0.02%)	Up	0
		Down	1		Down	1		Down	1
Number of peptides at T = 0*	2715								
Unstable peptides compared to T = 0	90 (3.3%)								

FC, intensity fold change of Tx versus T0; *identified in all three T, 0 samples

## Discussion

Fresh frozen tissue is increasingly used to study tumor biology by genomic, proteomic and cellular techniques. Clinical decisions are increasingly based on these, mostly genomic, assessments. Therefore, these tumor tissues should reliably reflect tumor biology and not be affected by variation related to the freezing procedure, including CIT [11, 12]. We studied the effect of tissue handling time on the (phospho) proteome and kinase activity profiles in human CRC tissue, taking the first biopsy in the operation room immediately after surgical tumor resection and found little effect when time to freezing is within 3 h.

Two different technologies were used in this study. NanoLC-MS/MS-based proteomics, providing an unbiased method for identifying approximately 10,000 phosphopeptides in their native cellular environment at natural abundances in a specific tumor sample, and two types of peptide microarrays, each containing 142 kinase substrate peptides which can be phosphorylated for measuring *ex-vivo* kinase-activity in the tumor sample. Since the principles of these two technologies, i.e. determining the presence of a phosphate on a protein or measuring the ability of kinases in the lysate to phosphorylate peptides, are very different, the results could not be compared.

Kinase activity profiling using peptide microarrays at 7 time points resulted in relatively small differences in phosphorylation signal caused by CIT in the five tumors tested for tyrosine and serine/threonine kinase activity. Despite these fluctuations in kinase activity with CIT, patient-specific tumor specific profiles were recovered.

Interestingly, the kinase activity profiles of patient 2, derived from a mucinous type tumor histology, are different from the other tumors analyzed. These differences may be tumor type dependent, but it is unknown whether or not this is a typical kinase activity profile of a mucinous type histology since only one mucinous tumor has been analyzed in the present study.

Nano LC/MS–MS, performed for three of these five patients, showed that the phosphoproteome is hardly influenced by leaving the tissue at room temperature up to 180 min. Of the 6044 phosphopeptides detected on average < 1% was significantly different comparing any two time points. Only two phosphopeptides (PKP3 and SRRM2) were significantly different in all time points compared including one peptides (PKP3) with a fold change > 2.

The current data on kinase activity and phosphoproteomic profiles hardly show common effects caused by CIT, but rather a patient-specific fluctuation over time. Both kinase activity and phosphoproteomic profiles may differ largely between the different patients since colorectal cancer is a very heterogeneous disease. Drivers of tumor development are reflected in the kinase profiles and may therefore be different as well. These tumor-specific features may also result in ischemia-induced differences between the tumor samples.

One-way ANOVA analysis of the phosphopeptides resulted in 90 (3.3%) peptides significantly changing with ischemic time, including MAPK1. This protein belongs to the MAP kinase family, known to play a role in stress response [15] and significant change of this protein has been shown previously within 60 min of CIT [8, 9, 12].

In the peptide microarray data, we analyzed phosphorylation of the MAPK substrate lamin B as readout for change in MAPK activity and found fluctuation in time, with highest activity at 30–60 min of CIT in most samples. However, the signals were low and did not show a significant consistent increase or decrease in signal intensity over three hours of time. In the nano LC/MS–MS data of the present study no significant change in signal intensity of the MAPK1 phosphopeptide was observed after 60 min of ischemic time, but after 120 and 180 min MAPK1 was decreased with a fold change of 10 and 7 (p = 0.02), respectively. Variation in MAPK signaling has been observed between tissue types or even between biopsy and resection specimen [12].

Tumor specific response to cold ischemia has been reported previously [9, 10]. Specific phosphorylation profiles of the tumors at baseline can occur in adjacent parts within a tumor and can influence the response to ischemia, contributing to differences in kinase activity measured between the tumor samples of each patient. Also, in the present study we observed differences in kinase activity profiles as the tumor of patient 1 showed a predominant increase in PTK activity and decrease in STK activity measured by the peptide array, while tumors from other patients show more fluctuation in increasing and decreasing sites.

In this study, the different samples from one patient originate from different locations in the tumor. Due to the heterogeneity of the tumor samples the tumor cell percentage of the different biopsies within one tumor sample vary. Tumor cell percentage is not necessarily correlated with signal intensities since kinase activity is not only determined by the tumor cells itself. Other components within the tumor, such as tumor stroma cells, may influence kinase activity. Also, samples with similar tumor percentage can differ in kinase activity profiles due to differences in underlying signaling routes.

Despite the heterogeneity of the tumor samples and the patient specific changes in response to ischemic time, unsupervised clustering showed that phosphorylation profiles are tumor specific rather than determined by CIT.

Previous studies showed contradictory results in stability of protein phosphorylation with ischemic time. Gündisch et al. used reverse phase protein array (RPPA) and LC–MS/MS to analyze changes in the phosphoproteome [11, 12]. To avoid heterogeneity of cancer samples they analyzed the phosphoproteome of normal intestine and liver tissue from patients with colorectal cancer and also of rat and mouse liver tissue samples. With RPPA no alterations of > twofold change were detected in rat and mouse liver samples after leaving the tissue samples at room temperature or on ice for 15 up to 360 min compared to time point 0, except for 1 protein, cytokeratin 18. Many phosphosites were stable up to 360 min of CIT and phosphoproteome changes due to prolonged cold ischemia detected with LC–MS/MS were unspecific [11]. RPPA and LC–MS/MS results of normal liver and intestine samples showed high interpatient and biological variability and global changes in phosphoprotein levels mainly occurring after 180 min [12]. In total 1684 phosphosites were detected in all replicates of mouse and rat liver tissue and 1812 sites were detected in the human intestine and liver samples which is comparable to the 1639 sites present in all samples in this study [11, 12].

Mertins et al*.* performed (phospho)proteomic analysis in patient-derived xenografts of human breast and ovarian cancer resulting in specific changes in a small subset of the phosphoproteome within 5 to 60 min of CIT while the global phosphoproteome is stable, comparable to the present study [10]. To accurately define CIT, blood vessels were not ligated prior to excision possibly explaining the differences in results compared to the present study and the study from Gundisch et al. in which time point 0 was defined after ligation of the blood vessels [10–12]. The present study was performed in a clinical setting, where the first biopsy (time point 0) was taken immediately after surgical tumor resection and immediately snap-frozen in liquid nitrogen in the operation room. The exact time between ligation of the vessels and tumor excision has not been recorded in our study.

Differences in tissue, human *versus* patient derived xenografts, might also contribute to changes of phosphosites with CIT since the time to remove the tumor tissue from the body is generally much longer in patients compared to mice. Several initial changes might already have occurred before the first measurements.

Espina et al. showed that protein kinases can remain active and reactive after surgical excision for at least 90 min, however time to start processing, defined as time point 0, varied between 4 and 40 min [8]. Also, David et al. showed rapid changes in protein phosphorylation status during and after surgical excision, influencing key proteins involved in colorectal cancer [16]. Gajadhar et al. specifically analyzed the stability of phosphotyrosine (pTyr) sites in ovarian and colon tumors and detected quantitative fluctuations of > twofold up to 50% of the pTyr sites within 5 min of cold ischemia especially in proteins involved in stress responses and MAPK signaling [9]. In the present study, the time between the first and second measurement after tumor excision was 30 min for protein kinase activity and 60 min for protein phosphorylation status. Therefore, rapid changes were missed. In addition, pTyr sites are suggested to be more susceptible to ischemia compared to pSer/pThr sites [9, 10] which were mainly identified in this study due to the abundance of serine/threonine phosphosites in the phosphoproteome.

In summary, this study shows that the majority of the phosphoproteome as well as the activity of protein kinases in colorectal cancer resection tissue is stable up to 180 min of CIT. However, kinase activity and the protein phosphorylation status may change during CIT and the differences in kinase activity and phosphoproteome may vary per tumor sample. These changes during CIT do not significantly obscure the tumor characteristics as the phosphoproteome and the kinase activity still reflect the tumor characteristics. Heterogeneity and tumor specific responses to ischemia may influence kinase activity profiles but this accounts for a minority of the phosphopeptides, when measured after at least 30 min of CIT. Despite these minimal differences, we do recommend standardized tissue collection procedures to minimize variability in measures of the phosphoproteome and protein kinase activity during CIT.

## Supplementary Information

**Additional file 1.** Coefficient of variation (CV) (Y-axis) as function of signal intensity (X-axis) for PTK for the 5 patients. The CVs of the seven timepoints (t = 0 and t = 30, t = 60, t = 90, t = 120, t = 150 and t = 180 min of CIT) are combined in one figure.

**Additional file 2.** Coefficient of variation (CV) (Y-axis) as function of signal intensity (X-axis) for STK for the 5 patients. The CVs of the seven timepoints (t = 0 and t = 30, t = 60, t = 90, t = 120, t = 150 and t = 180 min of CIT) are combined in one figure.

**Additional file 3: Figure S2.** Heatmap of protein tyrosine kinase (PTK) and serine/threonine kinase (STK) phosphorylation. Heatmap of 2log transformed mean signal intensities (X-axis) for PTK and STK activity profiles of the tumors of patient 1–5 for the time points 0, 30, 60, 90, 120, 150 and 180 min of CIT (Y-axis) for the peptides that passed quality control. Peptide names, Uniprot IDs, peptide sequences and position of tyrosine (Tyr), threonine (Thr) of serine (Ser) are given.

**Additional file 4: Table S1.** Peptides significantly changing with 180 min of CIT (p < 0.01, FC > 2), measured with a peptide microarray for patients 1–4 using a One-Way ANOVA test. In patient 5 none of the peptides were significantly changing with CIT. Peptides significantly altered, in patients 1–5 combined using a Mixed Model analysis, are shown in bold. UniProt Accession numbers, protein IDs and peptide sequence are given. **Table S2.** Phosphopeptides significantly changing with CIT (p < 0.01) between time points 0 and 60 min of CIT measured with MS-based proteomics. UniProt accession numbers, protein IDs, peptide sequences, fold changes (FC) and p-values are given. **Table S3.** Phosphopeptides significantly changing with CIT (p < 0.01) between time points 0 and 120 min of CIT measured with MS-based proteomics. UniProt accession numbers, protein IDs, peptide sequences, fold changes (FC) and p-values are given. **Table S4.** Phosphopeptides significantly changing with CIT (p < 0.01) between time points 0 and 180 min of CIT. UniProt accession numbers, protein IDs, peptide sequences, fold changes (FC) and p-values are given.

## Data Availability

Data is presented in the current study or in supplementary tables. Raw data of the current study is available from the corresponding author on reasonable request.
