# Supplementary figures and images for: Time dependent effect of cold ischemia on the phosphoproteome and protein kinase activity in fresh-frozen colorectal cancer tissue obtained from patients
Source: Clin Proteomics. 2021 Feb 18;18:8. doi: 10.1186/s12014-020-09306-6 (PMC7893972; doi:10.1186/s12014-020-09306-6)

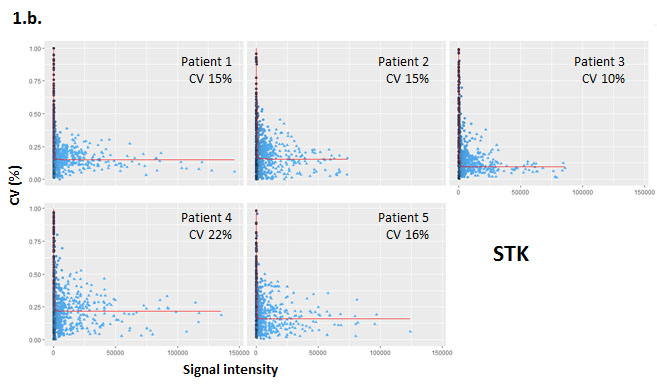

Supplement: Supplementary file 2 — Additional file 2. Coefficient of variation (CV) (Y-axis) as function of signal intensity (X-axis) for STK for the 5 patients. The CVs of the seven timepoints (t = 0 and t = 30, t = 60, t = 90, t = 120, t = 150 and t = 180 min of CIT) are combined in one figure. [file 12014_2020_9306_MOESM2_ESM.jpg]

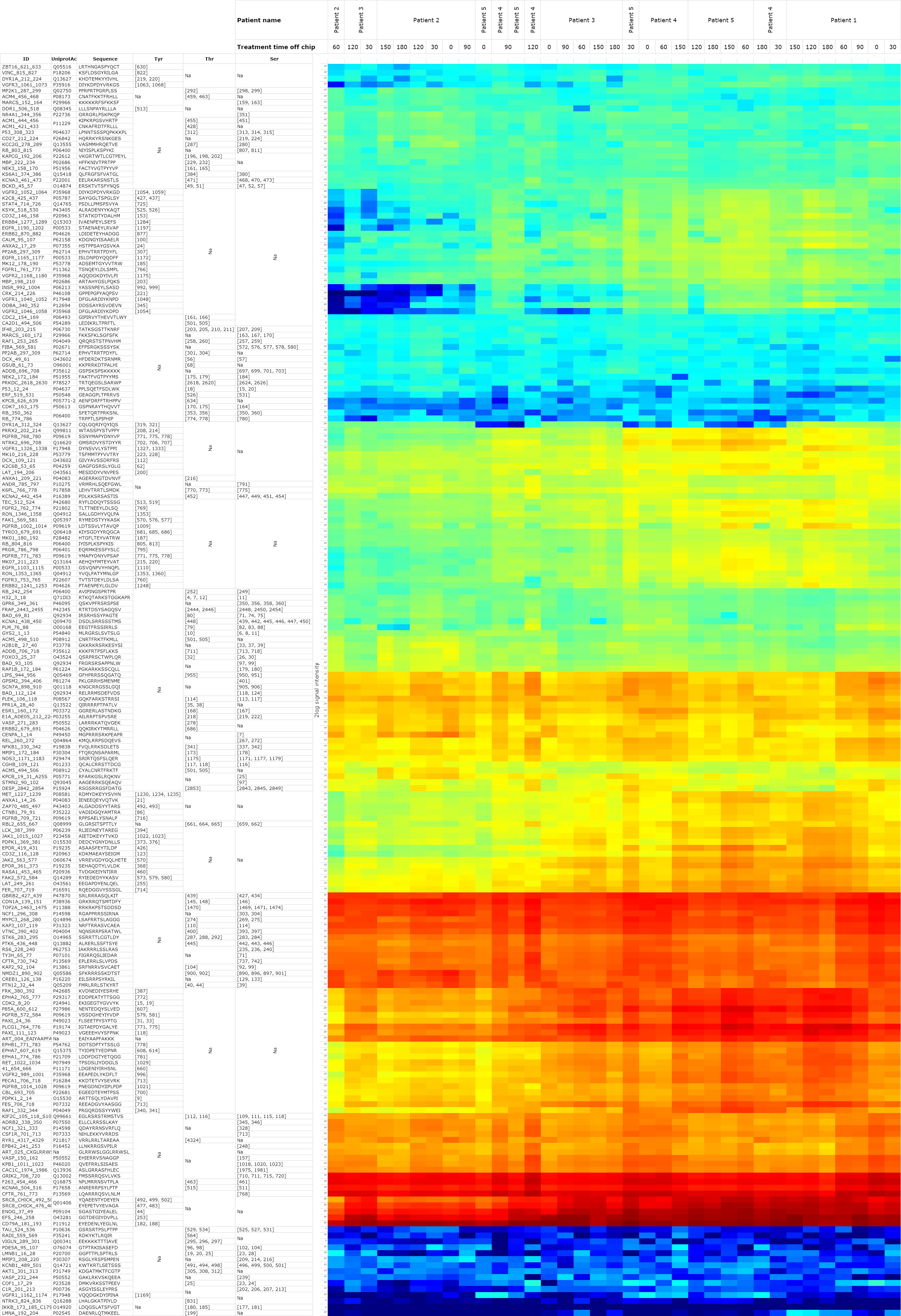

Supplement: Supplementary file 3 — Additional file 3: Figure S2. Heatmap of protein tyrosine kinase (PTK) and serine/threonine kinase (STK) phosphorylation. Heatmap of 2log transformed mean signal intensities (X-axis) for PTK and STK activity profiles of the tumors of patient 1–5 for the time points 0, 30, 60, 90, 120, 150 and 180 min of CIT (Y-axis) for the peptides that passed quality control. Peptide names, Uniprot IDs, peptide sequences and position of tyrosine (Tyr), threonine (Thr) of serine (Ser) are given. [file 12014_2020_9306_MOESM3_ESM.png]
