# Supplementary material for: Time dependent effect of cold ischemia on the phosphoproteome and protein kinase activity in fresh-frozen colorectal cancer tissue obtained from patients
Source: Clin Proteomics. 2021 Feb 18;18:8. doi: 10.1186/s12014-020-09306-6 (PMC7893972; doi:10.1186/s12014-020-09306-6)
Supplement: Supplementary file 4 — Additional file 4: Table S1. Peptides significantly changing with 180 min of CIT (p < 0.01, FC > 2), measured with a peptide microarray for patients 1–4 using a One-Way ANOVA test. In patient 5 none of the peptides were significantly changing with CIT. Peptides significantly altered, in patients 1–5 combined using a Mixed Model analysis, are shown in bold. UniProt Accession numbers, protein IDs and peptide sequence are given. Table S2. Phosphopeptides significantly changing with CIT (p < 0.01) between time points 0 and 60 min of CIT measured with MS-based proteomics. UniProt accession numbers, protein IDs, peptide sequences, fold changes (FC) and p-values are given. Table S3. Phosphopeptides significantly changing with CIT (p < 0.01) between time points 0 and 120 min of CIT measured with MS-based proteomics. UniProt accession numbers, protein IDs, peptide sequences, fold changes (FC) and p-values are given. Table S4. Phosphopeptides significantly changing with CIT (p < 0.01) between time points 0 and 180 min of CIT. UniProt accession numbers, protein IDs, peptide sequences, fold changes (FC) and p-values are given. [file 12014_2020_9306_MOESM4_ESM.docx]

**Tables**

Table S1. Peptides significantly changing with 180 minutes of CIT (p<0.01, FC>2), measured with a peptide microarray for patients 1-4 using a One-Way ANOVA test. In patient 5 none of the peptides were significantly changing with CIT. Peptides significantly altered, in patients 1-5 combined using a Mixed Model analysis, are shown in bold.

UniProt Accession numbers, protein IDs and peptide sequence are given.

|  | **UniProtAccession** | **Protein ID** | **Sequence** | **up/down** |
| --- | --- | --- | --- | --- |
| **Patient 1: PTK** | P27986 | P85A_600_612 | NENTEDQYSLVED | up |
|  | P29317 | EPHA2_765_777 | EDDPEATYTTSGG | up |
|  | P09619 | PGFRB_572_584 | VSSDGHEYIYVDP | up |
|  | P16284 | PECA1_706_718 | KKDTETVYSEVRK | up |
|  | P11171 | 41_654_666 | LDGENIYIRHSNL | up |
|  | P35968 | VGFR2_989_1001 | EEAPEDLYKDFLT | up |
|  | P21709 | EPHA1_774_786 | LDDFDGTYETQGG | up |
|  | P04049 | RAF1_332_344 | PRGQRDSSYYWEI | up |
|  | P22681 | CBL_693_705 | EGEEDTEYMTPSS | up |
|  | P54762 | EPHB1_771_783 | DDTSDPTYTSSLG | up |
|  | Q15375 | EPHA7_607_619 | TYIDPETYEDPNR | up |
|  | O43561 | LAT_249_261 | EEGAPDYENLQEL | up |
|  | O60674 | JAK2_563_577 | VRREVGDYGQLHETE | up |
|  | P20936 | RASA1_453_465 | TVDGKEIYNTIRR | up |
|  | P23458 | JAK1_1015_1027 | AIETDKEYYTVKD | up |
|  | P06239 | LCK_387_399 | RLIEDNEYTAREG | up |
|  | P04083 | ANXA1_14_26 | IENEEQEYVQTVK | up |
|  | P43403 | ZAP70_485_497 | ALGADDSYYTARS | up |
|  | P20963 | CD3Z_116_128 | KDKMAEAYSEIGM | up |
|  | Q04912 | RON_1346_1358 | SALLGDHYVQLPA | up |
|  | P21802 | FGFR2_762_774 | TLTTNEEYLDLSQ | up |
|  | **P09619** | **PGFRB_1002_1014** | **LDTSSVLYTAVQP** | **up** |
|  | Q05397 | FAK1_569_581 | RYMEDSTYYKASK | up |
|  | P00533 | EGFR_1103_1115 | GSVQNPVYHNQPL | up |
|  | Q13164 | MK07_211_223 | AEHQYFMTEYVAT | up |
|  | Q04912 | RON_1353_1365 | YVQLPATYMNLGP | up |
|  | **P07355** | **ANXA2_17_29** | **HSTPPSAYGSVKA** | **up** |
|  | P04626 | ERBB2_870_882 | LDIDETEYHADGG | up |
|  | P35968 | VGFR2_1052_1064 | DIYKDPDYVRKGD | up |
|  | **Q14765** | **STAT4_714_726** | **PSDLLPMSPSVYA** | **up** |
|  | P20963 | CD3Z_146_158 | STATKDTYDALHM | up |
|  | P12694 | ODBA_340_352 | DDSSAYRSVDEVN | up |
|  | P35968 | VGFR2_1046_1058 | DFGLARDIYKDPD | up |
| **Patient 1: STK** | P07550 | ADRB2_338_350 | ELLCLRRSSLKAY | down |
|  | P10275 | ANDR_785_797 | VRMRHLSQEFGWL | down |
|  | P04083 | ANXA1_209_221 | AGERRKGTDVNVF | down |
|  | NA | ART_025_CXGLRRWSLGGLRRWSL | GLRRWSLGGLRRWSL | down |
|  | Q92934 | BAD_112_124 | RELRRMSDEFVDS | down |
|  | Q92934 | BAD_69_81 | IRSRHSSYPAGTE | down |
|  | Q13936 | CAC1C_1974_1986 | ASLGRRASFHLEC | down |
|  | P38936 | CDN1A_139_151 | GRKRRQTSMTDFY | down |
|  | P13569 | CFTR_730_742 | EPLERRLSLVPDS | down |
|  | P13569 | CFTR_761_773 | LQARRRQSVLNLM | down |
|  | P01233 | CGHB_109_121 | QCALCRRSTTDCG | down |
|  | P23528 | COF1_17_29 | DMKVRKSSTPEEV | down |
|  | P16220 | CREB1_126_138 | EILSRRPSYRKIL | down |
|  | P07333 | CSF1R_701_713 | NIHLEKKYVRRDS | down |
|  | P15924 | DESP_2842_2854 | RSGSRRGSFDATG | down |
|  | P03255 | E1A_ADE05_212_224 | AILRRPTSPVSRE | down |
|  | P16452 | EPB42_241_253 | LLNKRRGSVPILR | down |
|  | P50548 | ERF_519_531 | GEAGGPLTPRRVS | down |
|  | P03372 | ESR1_160_172 | GGRERLASTNDKG | down |
|  | Q16875 | F263_454_466 | NPLMRRNSVTPLA | down |
|  | P42345 | FRAP_2443_2455 | RTRTDSYSAGQSV | down |
|  | P47870 | GBRB2_427_439 | SRLRRRASQLKIT | down |
|  | P46095 | GPR6_349_361 | QSKVPFRSRSPSE | down |
|  | P81274 | GPSM2_394_406 | PKLGRRHSMENME | down |
|  | Q13002 | GRIK2_708_720 | FMSSRRQSVLVKS | down |
|  | P33778 | H2B1B_ 27_40 | GKKRKRSRKESYSI | down |
|  | P17858 | K6PL_766_778 | LEHVTRRTLSMDK | down |
|  | P13861 | KAP2_92_104 | SRFNRRVSVCAET | down |
|  | P31323 | KAP3_107_119 | NRFTRRASVCAEA | down |
|  | Q09470 | KCNA1_438_450 | DSDLSRRSSSTMS | down |
|  | P16389 | KCNA2_442_454 | PDLKKSRSASTIS | down |
|  | P22001 | KCNA3_461_473 | EELRKARSNSTLS | down |
|  | P17658 | KCNA6_504_516 | ANRERRPSYLPTP | down |
|  | Q99661 | KIF2C_105_118_S106G | EGLRSRSTRMSTVS | down |
|  | P46020 | KPB1_1011_1023 | QVEFRRLSISAES | down |
|  | Q05469 | LIPS_944_956 | GFHPRRSSQGATQ | down |
|  | P29966 | MARCS_160_172 | FKKSFKLSGFSFK | down |
|  | P30304 | MPIP1_172_184 | FTQRQNSAPARML | down |
|  | Q14896 | MYPC3_268_280 | LSAFRRTSLAGGG | down |
|  | P14598 | NCF1_296_308 | RGAPPRRSSIRNA | down |
|  | P14598 | NCF1_321_333 | QDAYRRNSVRFLQ | down |
|  | P19838 | NFKB1_330_342 | FVQLRRKSDLETS | down |
|  | Q05586 | NMDZ1_890_902 | SFKRRRSSKDTST | down |
|  | P29474 | NOS3_1171_1183 | SRIRTQSFSLQER | down |
|  | **O00168** | **PLM_76_88** | **EEGTFRSSIRRLS** | **down** |
|  | Q13522 | PPR1A_28_40 | QIRRRRPTPATLV | down |
|  | Q13882 | PTK6_436_448 | ALRERLSSFTSYE | down |
|  | Q05209 | PTN12_32_44 | FMRLRRLSTKYRT | down |
|  | P04049 | RAF1_253_265 | QRQRSTSTPNVHM | down |
|  | P61224 | RAP1B_172_184 | PGKARKKSSCQLL | down |
|  | P06400 | RB_242_254 | AVIPINGSPRTPR | down |
|  | P06400 | RB_803_815 | NIYISPLKSPYKI | down |
|  | Q04864 | REL_260_272 | KMQLRRPSDQEVS | down |
|  | P62753 | RS6_228_240 | IAKRRRLSSLRAS | down |
|  | P21817 | RYR1_4317_4329 | VRRLRRLTAREAA | down |
|  | Q01118 | SCN7A_898_910 | KNGCRRGSSLGQI | down |
|  | O14965 | STK6_283_295 | SSRRTTLCGTLDY | down |
|  | Q93045 | STMN2_90_102 | AAGERRKSQEAQV | down |
|  | P11388 | TOP2A_1463_1475 | RRKRKPSTSDDSD | down |
|  | P07101 | TY3H_65_77 | FIGRRQSLIEDAR | down |
|  | P50552 | VASP_150_162 | EHIERRVSNAGGP | down |
|  | P04004 | VTNC_390_402 | NQNSRRPSRATWL | down |
| **Patient 2: PTK** | Q13627 | DYR1A_212_224 | KHDTEMKYYIVHL | down |
| **Patient 2: STK** | P49450 | CENPA_1_14 | MGPRRRSRKPEAPR | down |
| **Patient 3: STK** | P35612 | ADDB_696_708 | GSPSKSPSKKKKK | up |
| **Patient 4: STK** | P30307 | MPIP3_208_220 | RSGLYRSPSMPEN | up |

PTK: protein tyrosine kinase, STK: serine/threonine kinase, up: upregulated with CIT, down: downregulated with CIT

Table S2. Phosphopeptides significantly changing with CIT (p<0.01) between time points 0 and 60 minutes of CIT measured with MS-based proteomics. UniProt accession numbers, protein IDs, peptide sequences, fold changes (FC) and p-values are given.

| **UniProt accession** | **Protein ID** | **Sequence** | **FC** | **p-value** |
| --- | --- | --- | --- | --- |
| Q96TA1-2;Q96TA1 | FAM129B | GLLAQGLRPESPPPAGPLLNGAPAGESPQPK | -4.306 | 0.006 |
| Q8N4C8;Q8N4C8-2;Q8N4C8-3;Q8N4C8-4;Q8N4C8-5 | MINK1 | SDSVLPASHGHLPQAGSLER | -2.886 | 0.008 |
| Q96HP0;C9IZV6 | DOCK6 | SKSISSSNPDLAVAPGSVDDEVSR | -2.879 | 0.001 |
| **Q9Y446** | PKP3 | ADYDTLSLR | -2.604 | 0.009 |
| P15924;P15924-2 | DSP | SMSFQGIR | -2.386 | 0.005 |
| O60504-2;O60504 | SORBS3 | ADGGSPFLGRR | -1.862 | 0.006 |
| Q16666-3;Q16666-2;Q16666 | IFI16 | VSEEQTQPPSPAGAGMSTAMGR | -1.648 | 0.004 |
| Q96FF9;B5MBX0 | CDCA5 | APSPTKPLRR | -1.494 | 0.010 |
| P22466 | GAL | LLDLPAAASSEDIERS | -1.420 | 0.010 |
| Q8N3V7-2 |  | MRSPQPARPGSAAVPGAAFAPIPR | -1.246 | 0.004 |
| F5H4S9;F5H2X0;O95049-4;O95049;O95049-3;O95049-2 | TJP3 | HAQRSPEASQTDSPVESPR | 1.325 | 0.004 |
| Q9NQC3-5;Q9NQC3;Q9NQC3-2;Q9NQC3-4;F8WAM4 | RTN4 | MEDLDQSPLVSSSDSPPRPQPAFK | 1.408 | <0.001 |
| **Q9UQ35** | SRRM2 | TPAAAAAMNLASPR | 1.557 | 0.007 |
| Q14005-3;Q14005-2;Q14005 | IL16 | DPGVSESPPPGRQPNQK | 1.562 | 0.005 |
| P41236;Q6NXS1 | PPP1R2;PPP1R2P3 | IQEQESSGEEDSDLSPEEREK | 1.563 | 0.002 |
| Q15149-4 |  | KRTSSEDNLYLAVLR | 1.574 | 0.002 |
| Q96KC8 | DNAJC1 | QKDFDIAEQNESSDEESLRK | 1.648 | 0.005 |
| Q7Z5L9-2;Q7Z5L9;Q7Z5L9-3 | IRF2BP2 | NSNSPPSPSSMNQR | 1.812 | <0.001 |
| B7Z1L3;O00264 | PGRMC1 | EGEEPTVYSDEEEPKDESARK | 2.002 | 0.001 |
| P35749;P35749-2 | MYH11 | RVIENADGSEEETDTR | 2.050 | 0.001 |
| Q14980-2;Q14980;Q9BTE9 | NUMA1 | TQPDGTSVPGEPASPISQR | 2.092 | 0.007 |
| P51532-5;P51532-2;P51532-3;P51532-4;P51532;Q9HBD4 | SMARCA4 | IEKEDDSEGEESEEEEEGEEEGSESESR | 2.474 | 0.001 |
| B7Z1L3;O00264 | PGRMC1 | IVRGDQPAASGDSDDDEPPPLPR | 2.570 | 0.002 |
| Q9NYV4-2;Q9NYV4 | CDK12 | SLSRSPLPSR | 2.613 | 0.003 |
| °Q8TCJ2 | STT3B | ENPPVEDSSDEDDKRNQGNLYDK | 2.790 | 0.009 |
| Q9H6Y2;Q9H6Y2-2;G3V1J0 | WDR55 | EEGEDSMAQEEKEETGDDSD | 3.895 | 0.005 |
| °Q9Y5K6 | CD2AP | FNGGHSPTHSPEK | 5.326 | 0.007 |
| °Q92974-3;Q92974-2;Q92974;Q5VY93 | ARHGEF2 | EPALPLEPDSGGNTSPGVTANGEAR | 8.960 | 0.007 |

° overlapping between 0-60 and 0-120 minutes of CIT, ^+^ overlapping between 0-60 and 0-180 minutes of CIT, **bold**: overlapping between all different timepoints (0-60, 0-120 and 0-180 minutes of CIT).

Table S3. Phosphopeptides significantly changing with CIT (p<0.01) between time points 0 and 120 minutes of CIT measured with MS-based proteomics. UniProt accession numbers, protein IDs, peptide sequences, fold changes (FC) and p-values are given.

| \| **UniProt accession** \| **Protein ID** \| **Sequence** \| **FC** \| **p-value** \| \| --- \| --- \| --- \| --- \| --- \| \| Q9P1Y6-2;E9PJ24;F8WEF5;Q9P1Y6-3;Q9P1Y6 \| PHRF1 \| GAVAAEGASDTEREEPTESQGLAAR \| -8.298 \| 0.008 \| \| Q5T5P2;Q5T5P2-3;Q5T5P2-2;Q5T5P2-7;Q5T5P2-4;Q5T5P2-8;Q5T5P2-6;A6NLF3;F5H1Q3 \| KIAA1217 \| ISSLPVSRPISPSPSAILER \| -6.418 \| 0.005 \| \| P23528;E9PK25 \| CFL1 \| ASGVAVSDGVIK \| -5.486 \| 0.006 \| \| Q9NRY4 \| ARHGAP35 \| TSFSVGSDDELGPIR \| -4.856 \| 0.005 \| \| *O60716-21;O60716-19;O60716-13;O60716-11;O60716-5;O60716-3;O60716-24;O60716-23;O60716-16;O60716-18;O60716-15;O60716-17;O60716-10;O60716-8;O60716-9;O60716-7;C9JZR2;O60716-2;O60716;O60716-22;O60716-20;O60716-14;O60716-12;O60716-6;O60716-4;O60716-29;O60716-27;O60716-32;O60716-31;O60716-26;O60716-25;O60716-30;O60716-28 \| CTNND1 \| GSLASLDSLRK \| -4.356 \| 0.009 \| \| **Q9Y446** \| PKP3 \| ADYDTLSLRSLR \| -3.535 \| 0.001 \| \| P49585;C9JEJ2 \| PCYT1A \| MLQAISPKQSPSSSPTR \| -3.482 \| <0.001 \| \| Q15311 \| RALBP1 \| TGEPSPPHDILHEPPDVVSDDEKDHGK \| -3.376 \| 0.004 \| \| P85037;P85037-2 \| FOXK1 \| SGGLQTPECLSREGSPIPHDPEFGSK \| -3.260 \| 0.007 \| \| Q96PK6 \| RBM14 \| GQPGNAYDGAGQPSAAYLSMSQGAVANANSTPPPYER \| -2.769 \| 0.004 \| \| Q03701 \| CEBPZ \| SQLDDHPESDDEENFIDANDDEDMEKFTDADKETEIVK \| -2.670 \| 0.001 \| \| Q6WKZ4-2;E7EX40;Q6WKZ4-3;Q6WKZ4 \| RAB11FIP1 \| TPLSQSMSVLPTSKPEK \| -2.495 \| <0.001 \| \| A6NNK5;Q12888;F8VY86;Q12888-2 \| TP53BP1 \| MVIQGPSSPQGEAMVTDVLEDQKEGR \| -2.330 \| 0.002 \| \| B1AKC7;Q9UJF2-2 \| RASAL2 \| LEVPAERSPR \| -2.300 \| 0.009 \| \| Q14C86-3;Q14C86-5;Q14C86-2;Q14C86;Q14C86-6;F8W9S7;Q14C86-4 \| GAPVD1 \| SSDIVSSVR \| -2.245 \| 0.005 \| \| **Q9UQ35** \| SRRM2 \| MGQAPSQSLLPPAQDQPRSPVPSAFSDQSR \| -2.232 \| 0.010 \| \| Q9H7N4 \| SCAF1 \| REVLYDSEGLSGEER \| -1.860 \| 0.006 \| \| Q9Y2H2-4;Q9Y2H2 \| INPP5F \| SPSAGDVHILTGFAK \| -1.806 \| 0.003 \| \| O95297-4;O95297-2;O95297 \| MPZL1 \| KSPSDTEGLVK \| -1.778 \| 0.002 \| \| Q9NQT8;B4DGY5 \| KIF13B \| ERPDLEAPAPGSPFR \| -1.709 \| 0.001 \| \| P38432 \| COIL \| LIIESPSNTSSTEPA \| -1.434 \| 0.003 \| \| Q8TB72-2;B4E2B6;Q8TB72-3;Q8TB72 \| PUM2 \| GKASPFEEDQNR \| -1.375 \| 0.004 \| \| Q12802-4;Q12802;Q12802-2;A8MYJ1 \| AKAP13 \| SGSLDSELSVSPKR \| -1.371 \| 0.008 \| \| P26678 \| PLN \| RASTIEMPQQAR \| -1.342 \| 0.009 \| \| H7BXC4;Q5T5C0-2;Q5T5C0;Q5T5C0-3 \| STXBP5 \| SSSVTSIDKESR \| -1.326 \| 0.001 \| \| F5GZ78;P49023 \| PXN \| SSPGGQDEGGFMAQGK \| 1.342 \| 0.007 \| \| Q13459-2;Q13459;M0R0P8 \| MYO9B \| VQEKPDSPGGSTQIQR \| 1.402 \| 0.003 \| \| E9PNJ4;Q13586;E9PQJ4 \| STIM1 \| AEQSLHDLQER \| 1.426 \| 0.008 \| \| Q9HDC5 \| JPH1 \| KGTTPPRSPEASPK \| 1.558 \| 0.008 \| \| Q5T200-2;Q5T200 \| ZC3H13 \| SKGDSDISDEEAAQQSK \| 1.572 \| 0.001 \| \| E7EMW7;J3KMW7;O95071 \| UBR5 \| RISQSQPVR \| 1.607 \| 0.009 \| \| O14647-2;O14647 \| CHD2 \| HSDNPSEEGEVKDDGLEK \| 1.627 \| 0.005 \| \| *Q13501;Q13501-2;E7EMC7 \| SQSTM1 \| SSSQPSSCCSDPSKPGGNVEGATQSLAEQMR \| 1.932 \| 0.008 \| \| P27824;B4DGP8;B4E2T8 \| CANX \| QKSDAEEDGGTVSQEEEDR \| 2.010 \| 0.009 \| \| O14974-5;O14974-4;O14974-3;O14974-2;O14974 \| PPP1R12A \| DKKSPLIESTANMDNNQSQK \| 2.057 \| 0.001 \| \| Q6ZRV2 \| FAM83H \| RGSPTTGFIEQK \| 2.159 \| 0.003 \| \| Q7Z3C6-2;Q7Z3C6 \| ATG9A \| RESDESGESAPDEGGEGAR \| 2.251 \| <0.001 \| \| P05114;F2Z2W6;F2Z2Y5;H7BXJ5 \| HMGN1 \| KVSSAEGAAKEEPK \| 2.288 \| <0.001 \| \| G3V578;Q9UN36-4;Q9UN36-5;Q9UN36-2;Q9UN36-3;B4DE86;Q9UN36 \| NDRG2 \| TLSQSSESGTLSSGPPGHTMEVSC \| 2.663 \| 0.008 \| \| P08240-2;P08240 \| SRPR \| GTGSGGQLQDLDCSSSDDEGAAQNSTKPSATK \| 2.697 \| 0.001 \| \| O60271-5;O60271-4;O60271-2;O60271-9;O60271-3;O60271 \| SPAG9 \| SASQSSLDKLDQELK \| 2.760 \| 0.007 \| \| °Q92974-3;Q92974-2;Q92974;Q5VY93 \| ARHGEF2 \| SESLESPRGER \| 3.230 \| 0.003 \| \| Q9UHB6;Q9UHB6-4;F8VQE1;Q9UHB6-2;F8VS07;Q9UHB6-3;F8VRN8 \| LIMA1 \| ASSLSESSPPK \| 3.314 \| 0.008 \| \| °Q8TCJ2 \| STT3B \| ENPPVEDSSDEDDKRNQGNLYDK \| 3.598 \| 0.009 \| \| °P41236;Q6NXS1 \| PPP1R2;PPP1R2P3 \| IQEQESSGEEDSDLSPEER \| 3.759 \| 0.006 \| \| *Q9UQ35;Q9UQ35-2 \| SRRM2 \| AQSGSDSSPEPKAPAPR \| 3.965 \| 0.007 \| \| Q5VZL5-2;Q5VZL5-4;Q5VZL5;Q5VZL5-3 \| ZMYM4 \| AKSEDSDVELSD \| 6.774 \| 0.004 \| \| °Q9Y5K6 \| CD2AP \| FNGGHSPTHSPEK \| 9.953 \| 0.009 \| |  |  |  |  |
| --- | --- | --- | --- | --- | --- | --- | --- | --- | --- | --- | --- | --- | --- | --- | --- | --- | --- | --- | --- | --- | --- | --- | --- | --- | --- | --- | --- | --- | --- | --- | --- | --- | --- | --- | --- | --- | --- | --- | --- | --- | --- | --- | --- | --- | --- | --- | --- | --- | --- | --- | --- | --- | --- | --- | --- | --- | --- | --- | --- | --- | --- | --- | --- | --- | --- | --- | --- | --- | --- | --- | --- | --- | --- | --- | --- | --- | --- | --- | --- | --- | --- | --- | --- | --- | --- | --- | --- | --- | --- | --- | --- | --- | --- | --- | --- | --- | --- | --- | --- | --- | --- | --- | --- | --- | --- | --- | --- | --- | --- | --- | --- | --- | --- | --- | --- | --- | --- | --- | --- | --- | --- | --- | --- | --- | --- | --- | --- | --- | --- | --- | --- | --- | --- | --- | --- | --- | --- | --- | --- | --- | --- | --- | --- | --- | --- | --- | --- | --- | --- | --- | --- | --- | --- | --- | --- | --- | --- | --- | --- | --- | --- | --- | --- | --- | --- | --- | --- | --- | --- | --- | --- | --- | --- | --- | --- | --- | --- | --- | --- | --- | --- | --- | --- | --- | --- | --- | --- | --- | --- | --- | --- | --- | --- | --- | --- | --- | --- | --- | --- | --- | --- | --- | --- | --- | --- | --- | --- | --- | --- | --- | --- | --- | --- | --- | --- | --- | --- | --- | --- | --- | --- | --- | --- | --- | --- | --- | --- | --- | --- | --- | --- | --- | --- | --- | --- | --- | --- | --- | --- | --- | --- | --- | --- | --- | --- | --- | --- | --- | --- |
|  |  |  |  |  |

° overlapping between 0-60 and 0-120 minutes of CIT, *overlapping between 0-120 and 0-180 minutes of CIT, **bold**: overlapping between all different timepoints (0-60, 0-120 and 0-180 minutes of CIT).

Table S4. Phosphopeptides significantly changing with CIT (p<0.01) between time points 0 and 180 minutes of CIT. UniProt accession numbers, protein IDs, peptide sequences, fold changes (FC) and p-values are given.

| \| **UniProt accession** \| **Protein ID** \| **Sequence** \| **FC** \| **p-value** \| \| --- \| --- \| --- \| --- \| --- \| \| Q13557-12;Q13557-8;Q13557;Q13557-10;E9PBG7;Q13557-6;Q13557-11;E9PF82;Q13557-9;Q13557-5;Q13557-3;Q13557-4 \| CAMK2D \| NFSAAKSLLK \| -4.603 \| 0.004 \| \| B4DKL4;Q86X29-5;Q86X29-4;Q86X29;Q86X29-3;Q86X29-2 \| LSR \| ARSVDALDDLTPPSTAESGSR \| -4.411 \| 0.001 \| \| **Q9Y446** \| PKP3 \| SAVDLSCSR \| -4.311 \| 0.009 \| \| *O60716-21;O60716-19;O60716-13;O60716-11;O60716-5;O60716-3;O60716-24;O60716-23;O60716-16;O60716-18;O60716-15;O60716-17;O60716-10;O60716-8;O60716-9;O60716-7;C9JZR2;O60716-2;O60716;O60716-22;O60716-20;O60716-14;O60716-12;O60716-6;O60716-4;O60716-29;O60716-27;O60716-32;O60716-31;O60716-26;O60716-25;O60716-30;O60716-28 \| CTNND1 \| GSLASLDSLR \| -3.593 \| 0.005 \| \| P10451-4;P10451;P10451-3;P10451-5;P10451-2 \| SPP1 \| ANDESNEHSDVIDSQELSKVSR \| -3.560 \| 0.006 \| \| Q8IVT2 \| C19orf21 \| RALSSDSILSPAPDAR \| -3.166 \| 0.001 \| \| P10451-4;P10451;P10451-3;P10451-5;P10451-2;CON__P31096 \| SPP1 \| ISHELDSASSEVN \| -2.343 \| 0.007 \| \| E7EV56;Q15154-2;E7ETA6;Q15154;Q15154-3;E9PGW9 \| PCM1 \| VTNDISPESSPGVGR \| -2.202 \| 0.010 \| \| Q5VT25-3;Q5VT25-4;F5H5N0;Q5VT25-5;Q5VT25;Q5VT25-2;Q5VT25-6 \| CDC42BPA \| SLSLESTDR \| -1.953 \| 0.006 \| \| P16144-4;P16144-2;P16144-3;P16144 \| ITGB4 \| MDFAFPGSTNSLHR \| -1.940 \| 0.005 \| \| Q5C9Z4 \| NOM1 \| VRFAEDEEKSENSSEDGDITDK \| -1.875 \| 0.007 \| \| O60678 \| PRMT3 \| GAVENEEDLPELSDSGDEAAWEDEDDADLPHGK \| -1.835 \| 0.008 \| \| B4DQA8;E9PCW1 \| GOSR1 \| RDSSDTTPLLNGSSQDR \| -1.805 \| 0.003 \| \| Q6PKG0;Q6PKG0-3 \| LARP1 \| AVTPVPTKTEEVSNLK \| -1.719 \| 0.005 \| \| E7EV54;F8WDZ1;Q8IZL8;I3L3A8;C9JFV4;Q8IZL8-2 \| PELP1 \| GSPDGSLQTGKPSAPK \| -1.674 \| 0.004 \| \| Q8IYB1 \| MB21D2 \| RGSTTSIPSPQSDGGDPNQPDDR \| -1.586 \| 0.004 \| \| Q99549;Q99549-2 \| MPHOSPH8 \| GAEAFGDSEEDGEDVFEVEK \| -1.416 \| 0.003 \| \| Q9NQG6;B0QY95 \| SMCR7L \| SLQTLPTDSSTFDTDTFCPPRPKPVAR \| -1.408 \| 0.003 \| \| F5H2E2;F5GWM7;Q7Z460-2;B7ZLX3;F5GWS0;Q7Z460-3;F8WA11;Q7Z460 \| CLASP1 \| NSSNTSVGSPSNTIGR \| -1.252 \| 0.008 \| \| Q9UBC2-3;Q9UBC2;Q9UBC2-2;M0R2S2;Q9UBC2-4;M0R165 \| EPS15L1 \| STPSHGSVSSLNSTGSLSPK \| -1.187 \| 0.005 \| \| F5H629;B8ZZU6;E9PBF9;P15336-5;P15336;P15336-2;P15336-4 \| ATF2 \| TQSEESRPQSLQQPATSTTETPASPAHTTPQTQSTSGR \| -1.127 \| 0.001 \| \| P55201-4;P55201-3;P55201;P55201-2 \| BRPF1 \| GSLTPHPAACDKDGQTDSAAEESSSQETSK \| 1.085 \| 0.007 \| \| Q8TDB6 \| DTX3L \| EGHETPMDIDSDDSK \| 1.214 \| 0.006 \| \| Q8N201;A4D212 \| INTS1;DKFZP586J0619 \| SSPEQPIGQGR \| 1.245 \| 0.008 \| \| E9PNJ4;Q13586 \| STIM1 \| SHSPSSPDPDTPSPVGDSR \| 1.263 \| 0.002 \| \| Q8WVT3 \| TRAPPC12 \| SPSFGGASEASAR \| 1.354 \| 0.006 \| \| Q5VZ89-6;Q5VZ89;R4GNB2;R4GN35;Q5VZ89-5 \| DENND4C \| HPTGNSITKSPPLMAK \| 1.378 \| 0.004 \| \| P20042 \| EIF2S2 \| SGDEMIFDPTMSK \| 1.473 \| 0.006 \| \| O95400 \| CD2BP2 \| HSLDSDEEEDDDDGGSSK \| 1.554 \| 0.004 \| \| Q86VM9;E7ERS3;Q86VM9-2 \| ZC3H18 \| GPSQEEEDNHSDEEDRASEPKSQDQDSEVNELSR \| 1.562 \| 0.008 \| \| Q9UDY2-3;Q9UDY2;Q9UDY2-7;Q9UDY2-6;Q9UDY2-4;Q9UDY2-2;Q9UDY2-5 \| TJP2 \| AYSPEYR \| 1.603 \| 0.005 \| \| **Q9UQ35** \| SRRM2 \| SRTPPSAPSQSR \| 1.631 \| 0.002 \| \| O00499-6;O00499-11;O00499-2;O00499;O00499-9;O00499-10;O00499-7;O00499-8;O00499-4;O00499-3;O00499-5 \| BIN1 \| GNKSPSPPDGSPAATPEIR \| 1.799 \| 0.002 \| \| Q9Y2D5;Q9Y2D5-5;Q9Y2D5-7;Q9Y2D5-6;Q9Y2D5-4 \| AKAP2 \| GQKSPGALETPSAAGSQGNTASQGK \| 1.809 \| 0.009 \| \| Q5SSJ5-3;Q5SSJ5-2;Q5SSJ5 \| HP1BP3 \| KEPDDSRDEDEDEDESSEEDSEDEEPPPK \| 1.867 \| 0.001 \| \| Q5T757;Q05519-2;Q05519 \| SRSF11 \| VNGDDHHEEDMDMSD \| 1.885 \| <0.001 \| \| ^+^B7Z1L3;O00264 \| PGRMC1 \| EGEEPTVYSDEEEPKDESAR \| 1.991 \| 0.002 \| \| Q9UQ35;Q9UQ35-2 \| SRRM2 \| SRSPSSPELNNK \| 2.006 \| 0.002 \| \| O43432;O43432-3;F5H564;F5H8J4;Q504Z1 \| EIF4G3 \| LDFIESDSPCSSEALSK \| 2.139 \| 0.007 \| \| Q14847;Q14847-2;B4DGQ0 \| LASP1 \| MGPSGGEGMEPERRDSQDGSSYR \| 2.143 \| 0.004 \| \| Q9HB58-2;Q9HB58-5;G5E9C0;Q9HB58-3;Q9HB58-7;Q9HB58;Q9HB58-6 \| SP110 \| DKEDPQEMPHSPLGSMPEIR \| 2.479 \| 0.003 \| \| O60565 \| GREM1 \| GTAMPGEEVLESSQEALHVTER \| 2.490 \| 0.007 \| \| Q15648 \| MED1 \| SYQNSPSSDDGIRPLPEYSTEK \| 2.536 \| 0.010 \| \| Q96A00-2;Q96A00;K7EJB8;K7EMN0 \| PPP1R14A \| ARGPGGSPGGLQK \| 2.646 \| 0.004 \| \| D6RCP9;P27707;D6RFG8 \| DCK \| WCNVQSTQDEFEELTMSQK \| 2.764 \| 0.005 \| \| H3BQZ7;Q1KMD3 \| hCG_2044799;HNRNPUL2 \| EEDEPEERSGDETPGSEVPGDK \| 3.490 \| 0.003 \| \| *Q13501;Q13501-2;E7EMC7 \| SQSTM1 \| SSSQPSSCCSDPSKPGGNVEGATQSLAEQMR \| 3.562 \| 0.009 \| \| *Q9UQ35;Q9UQ35-2 \| SRRM2 \| SSRSSPELTR \| 3.800 \| 0.003 \| \| Q8WWQ0 \| PHIP \| TAFYNEDDSEEEQR \| 4.749 \| 0.007 \| \| P21333-2;P21333;Q5HY54;E9PHF0 \| FLNA \| IPEISIQDMTAQVTSPSGK \| 5.042 \| <0.001 \| \| P19338 \| NCL \| EVEEDSEDEEMSEDEEDDSSGEEVVIPQKK \| 6.403 \| 0.006 \| \| Q15746-4;Q15746-5;Q15746-2;Q15746-3;Q15746-6;Q15746;Q15746-7;B4DUE3;Q6P2N0;Q15746-8 \| MYLK \| KSSTGSPTSPLNAEK \| 7.493 \| 0.008 \| |  |  |  |  |
| --- | --- | --- | --- | --- | --- | --- | --- | --- | --- | --- | --- | --- | --- | --- | --- | --- | --- | --- | --- | --- | --- | --- | --- | --- | --- | --- | --- | --- | --- | --- | --- | --- | --- | --- | --- | --- | --- | --- | --- | --- | --- | --- | --- | --- | --- | --- | --- | --- | --- | --- | --- | --- | --- | --- | --- | --- | --- | --- | --- | --- | --- | --- | --- | --- | --- | --- | --- | --- | --- | --- | --- | --- | --- | --- | --- | --- | --- | --- | --- | --- | --- | --- | --- | --- | --- | --- | --- | --- | --- | --- | --- | --- | --- | --- | --- | --- | --- | --- | --- | --- | --- | --- | --- | --- | --- | --- | --- | --- | --- | --- | --- | --- | --- | --- | --- | --- | --- | --- | --- | --- | --- | --- | --- | --- | --- | --- | --- | --- | --- | --- | --- | --- | --- | --- | --- | --- | --- | --- | --- | --- | --- | --- | --- | --- | --- | --- | --- | --- | --- | --- | --- | --- | --- | --- | --- | --- | --- | --- | --- | --- | --- | --- | --- | --- | --- | --- | --- | --- | --- | --- | --- | --- | --- | --- | --- | --- | --- | --- | --- | --- | --- | --- | --- | --- | --- | --- | --- | --- | --- | --- | --- | --- | --- | --- | --- | --- | --- | --- | --- | --- | --- | --- | --- | --- | --- | --- | --- | --- | --- | --- | --- | --- | --- | --- | --- | --- | --- | --- | --- | --- | --- | --- | --- | --- | --- | --- | --- | --- | --- | --- | --- | --- | --- | --- | --- | --- | --- | --- | --- | --- | --- | --- | --- | --- | --- | --- | --- | --- | --- | --- | --- | --- | --- | --- | --- | --- | --- | --- | --- | --- | --- | --- | --- | --- | --- | --- | --- | --- | --- |
|  |  |  |  |  |
| ^+^ overlapping between 0-60 and 0-180 minutes of CIT, *overlapping between 0-120 and 0-180 minutes of CIT, **bold**: overlapping between all different timepoints (0-16, 0-120 and 0-180 minutes of CIT). |  |  |  |  |
|  |  |  |  |  |
|  |  |  |  |  |
